# Rasch analysis of the Herth Hope Index in cancer patients

**DOI:** 10.1186/s12955-018-1025-5

**Published:** 2018-10-03

**Authors:** Tone Rustøen, Anners Lerdal, Caryl Gay, Anders Kottorp

**Affiliations:** 1 Department of Nursing Science, Institute of Health and Society, Faculty of Medicine, University of Oslo, Oslo, Norway; 2 Department of Research and Development, Division of Emergencies and Critical Care, Oslo University Hospital, 0424 Oslo, Norway; 3 Department for Patient Safety and Research, Lovisenberg Diakonale Hospital, Oslo, Norway; 4 Department of Family Health Care Nursing, School of Nursing, University of California, San Francisco, CA USA; 5 Faculty of Health and Society, Malmö University, Malmö, Sweden

## Abstract

**Background:**

The concept of hope has been measured using the Herth Hope Index (HHI) in different samples, but varying factor structures comprising different items from the HHI have been reported. Therefore, further testing with regard to the dimensionality of the instrument is recommended. Rasch modeling can be used to evaluate validity evidence of an instrument’s underlying structure, to identify items with poor fit to the rest of the scale, and to identify items that perform inconsistently across groups. The aim of this study was to assess the HHI’s psychometric properties in a sample of cancer patients using a Rasch model. Adult oncology outpatients (*n* = 167) with pain from bone metastasis were included, and medical records were reviewed for disease and treatment information. Patients completed the 12-item HHI, which measures various dimensions of hope using a 4-point Likert scale that ranges from 1 (strongly disagree) to 4 (strongly agree). The internal scale validity, person response validity, unidimensionality, and uniform differential item functioning were evaluated by applying a Rasch rating scale model.

**Results:**

Five (42%) of the twelve items (#3, #4, #5, #6 and #7) did not meet the criterion set for item goodness-of-fit. After removing these 5 items, the resulting 7-item scale demonstrated acceptable item fit to the model, acceptable unidimensionality (52.6% of the variance explained), acceptable person goodness-of-fit, adequate separation, and no differential item function.

**Conclusion:**

A 7-item version of the HHI had better psychometric properties than the original 12-item version among patients with cancer-related pain.

**Trial registration:**

The protocol ID is 158,707/V10 and it was registered on ClinicalTrials.gov as NCT00760305. Registered September 25, 2008.

## Background

Hope has been measured in many different patient samples [1–5], in family caregivers [6, 7] and in the general population [8]. Hope is described as an important phenomenon for patients in different phases of their disease [2, 9] as well as for their quality of life [10, 11]. Hope is considered to be an effective coping strategy for both patients [12, 13] and family caregivers [7] in demanding situations in life. One commonly used measure of hope is the Herth Hope Index (HHI) [14]. The HHI is a 12-item instrument designed to measure a global, non-time oriented sense of hope. The HHI is based on a definition of hope that describes it as a multidimensional life force characterized by a confident yet uncertain expectation of achieving a future good, which to the hoping person is realistically possible and personally significant [15]. The three dimensions of the HHI are defined to be: temporality and future, positive readiness and expectancy, and interconnectedness [14].

The HHI was developed from the 30-item Herth Hope Scale (HHS) [16]. The HHS was reduced from 30 to 12 items to make a shorter scale and still capture the multi-dimensionality of hope as presented in the HHS [14, 16]. Herth did a factor analysis of adults in clinical settings and found a three-factor solution explaining 61% of the variance in HHI scores [14]. All 12 HHI items had a significant loading on one of the three factors corresponding to the Herth Hope Scale. Factor 1 (Inner sense of temporality and future) consisted of items #1, #2, #6 and #11, Factor 2 (Inner positive readiness and expectancy) consisted of items #4, #7, #10 and #12, and Factor 3 (Interconnectedness with self and others) consisted of items #3, #5, #8 and #9.

After Herth’s factor analysis, 8 studies further examined the factor structure of the HHI using various approaches [6, 8, 17–22] (Table 1). Interestingly, in these eight studies, one [20, 21], two [6, 8, 17, 19, 22] or three factors [18] were identified. These differing results may be due to the fact that the studies not only used different analytic approaches, but also used the HHI in different types of samples and in different countries. Still, the findings indicate that there is no empirically confirmed construction of the phenomenon of hope as measured by the HHI.

**Table 1:** An overview of studies that have performed a factor analyses on Herth Hope Index

Authors & Year	Sample (Country)	HHI Mean (SD)	Cronbach’s alpha	Test-retest reliability	Factor analysis	Criterion/convergent validity	Discriminant validity
Herth 1992	172 adults acute, chronic and terminally ill (US)	–	0.97	0.91	3 factors (MLFA) (61% variance) Factor 1: 1,2,6,11 Factor 2: 4,7,10,12 Factor 3: 3,5,8,9	Herth Hope Scale *r* = 0.92 Existential Well-being Scale *r* = 0.84 Nowotny Hope Scale *r* = 0.81	Hopelessness Scale *r* = −0.73
Benzein & Berg 2003	85 adults, 40 patients in palliative care & 45 family caregivers (Sweden)	–	0.88	–	2 factors (PCA) (56% variance) Factor 1: 1–3,6–12 Factor 2: 4,5	Miller Hope Scale *r* = 0.82	Hopelessness *r* = −0.69
Wahl et al. 2004	1893 - general population (Norway)	36.7 (4.2)	0.81	–	2 factors (MLFA) (38% variance) Factor 1: 1,2,4,5,7–12 Factor 2: 3,6	Quality of Life *r* = 0.48	Fatigue severity *r* = −0.30
Phillips –Salimi et al. 2007	Adolescents/children with cancer, 127 in treatment, 74 newly diagnosed (US)	–	0.84 in treatment, 0.78 newly diagnosed	–	1 factor (CFA)	Self-esteem *r* = 0.62 Self-confidence *r* = 0.57 Self-transcendence *r* = 0.58 Quality of life *r* = 0.33	Uncertainty in illness *r* = −0.36 Symptom dis-tress *r* = − 0.22
Van Gestel-Timmermans et al. 2010	341 severe mental illness (Netherlands)	–	0.84	0.79	2 factors (PCA) (47% variance) Factor 1: 1–3,6,10,12 Factor 2: 4,5,7-9,11	Self-efficacy *r* = 0.72 Quality of Life *r* = 0.56 Mental health *r* = 0.59	Loneliness *r* = −0.47
Chan et al. 2011	120 patients with heart failure (China)	–	0.89	0.86	3 factors (CFA) Factor 1:1,2,6,11 Factor 2: 4,7,10,12 Factor 3: 3,5,8,9	Self-esteem *r* = 0.40	Depression *r* = −0.40
Ripamonti et al. 2012	266 patients with non-advanced cancer (Italy)	–	0.84	0.64	1 factor (CFA)	Spiritual Well-being *r* = 0.69,	Anxiety-Depression *r* = −0.51, Fatigue *r* = − 0.30
Haugan et al. 2013	202 cognitively intact nursing home patients (Norway)	35.1 (4.2)	0.76	–	2 factors (CFA) 11 items (exclude #6) (42% variance) Factor 1: 1–5,10–12 Factor 2: 7–9	Self-transcendence r = 0.59 Purpose in life *r* = 0.65 Spiritual well-being r = 0.72	Depression *r* = −0.22
Hunsaker et al. 2016	51 family members and 45 cognitive impaired patients (US)	38.8 (5.2) for family, 39.7 (5.2) for patients	0.85	–	2 factors (EFA) (51% variance) Factor 1: 2,3,7,9,10,12 Factor 2: 1,4-6,8,11	Satisfaction with social support *r* = 0.37	Depression *r* = −.21

*Abbreviations*: *CFA* confirmatory factor analysis, *EFA* exploratory factor analysis, *HHI* Herth Hope Index, *MLFA* multilevel factor analysis, *PCA* principal components analysis

The psychometric properties of the HHI were evaluated in a representative sample of the Norwegian population [8], and a factor analysis resulted in a two-factor solution, which explained 38% of the variance. One factor consisted of four items, including #1, #2, #3 and #6. The other six items loaded on the second factor. These findings suggested that positively worded items cluster together on a dominant factor as both items #3 and #6 are negatively worded and items #1 and #2 loaded on both factors [8]. The two factors were not named in this study, but three of four items in one factor were future oriented (positive outlook on life, presence of goals and scared about the future). The other factor comprised the rest of the items.

Benzein and Berg [17] examined the factor structure of the HHI in Swedish palliative cancer patients. They also found a two-factor solution explaining 56% of the variance. One factor consisted of two items, #4 and #5, and they named it “Religiosity”. The second factor consisted of the other 10 items and was named “Reconciliation with life situation”. With only two items, the first factor explained only 9.4% of the variance. One can also question whether item #4 (I can see a light in the tunnel) reflects religiosity.

In a Dutch study with patients with severe mental health problems, a two-factor solution was found explaining 47% of the variance [22]. The first factor (view on life and future) consisted of items #1, #2, #3, #6, #10 and #12, while the second factor (self-confidence and inner strength) consisted of the other six items.

In a study describing the psychometric properties in cognitively intact Norwegian nursing home patients, Haugan and colleagues described different factor solutions of the HHI [19]. A two-factor model comprising 11 items (excluding item #6) resulted in the best model fit. One factor was named “Temporality and readiness” and the other “Interconnect”. This model explained 41.6% of the variance. The factor structure of the HHI was examined in US patients with cognitive impairment and their family caregivers [6]. They found a two-factor solution explaining 51.4% of the variance. Factor 1 comprised items #2, #3, #7, #9, #10 and #12, while factor 2 comprised items #1, #4, #5, #6, #8, and #11. Factor 2 explained only 6.05% of the variance.

In Chinese patients with heart failure [18], a confirmatory factor analysis yielded the same three-factor solution as Herth found in her original work [14]. The percent of explained variance was not reported in the Chan et al. study. In Italian patients with solid and hematological malignancies on active treatment [21], a one-factor solution was found to have the best fit.

Taken together, previous studies using classical test theory approaches found varying factor structures comprising different items from the HHI. The measureable concept of hope seems to be influenced by various aspects, including both methodological approaches as well as sample-dependent characteristics. Therefore, further testing with regard to the dimensionality of the instrument is recommended. Approaches based on item response theory (IRT), such as Rasch modeling, have certain advantages over classical test theory and can be used to more fully evaluate validity evidence of an instrument’s underlying structure, to identify items with poor fit to the rest of the scale, and to identify items that perform inconsistently across groups. Another limitation of these prior studies using the HHI is that they are based on an assumption that the scores generated from the HHI can be treated as interval measures. The consequences of treating ordinal data as interval have been previously described [23, 24], and specifically in relation to the use of factor analysis [25, 26]. As a Rasch model is better modeled to use ordinal data for evaluation of a scale’s psychometric properties [27], and as it has not been used in any of the pervious validation studies of the HHI, it was the methodological choice for this study. Thus, the aim of this study was to assess the HHI’s psychometric properties in a sample of cancer patients from Norway using a Rasch modeling approach.

## Methods

### Sample and settings

The patients and their family caregivers in this study were recruited for participation in a randomized controlled trial about pain management. Only baseline data from the patients are included in this analysis. Details about the recruitment procedure and the samples are described in detail elsewhere [28]. Adult oncology outpatients with pain from bone metastasis (*n* = 179) were included from a university-based cancer center. All were outpatients who were able to read, write, and understand Norwegian. Because this study was about pain management, all patients had an average pain intensity score of 2.5 or greater on a 0–10 numeric rating scale and radiographic evidence of bone metastasis. To ensure satisfactory physical functioning, only patients with a Karnofsky Performance Status (KPS) score of 50 or greater were eligible. The Regional Committee for Medical and Health Research Ethics approved the study. The protocol was registered on ClinicalTrials.gov as NCT00760305.

### Measures

#### Demographic and clinical data

Medical records were reviewed for disease and treatment information, including cancer diagnosis, treatment and radiographic evidence of bone metastasis. Patients completed a demographic questionnaire about age, gender, living arrangements, education and employment status. The nurse who recruited the patients also completed the KPS [29] to evaluate each patient’s physical functioning.

#### Herth Hope index (HHI)

All patients completed the HHI after recruitment into the study. The HHI measures various dimensions of hope using a 4-point Likert scale that ranges from 1 (strongly disagree) to 4 (strongly agree) with items #3 and #6 reverse-coded. The scale has one global score that ranges from 12 to 48, as well as single-item scores that range from 1 to 4 [14]. A higher score denotes higher levels of hope. In addition to the evidence of its validity, its reliability has also been evaluated and found to be satisfactory. Both internal consistency [6, 8, 14, 17–22] and test-retest correlations [14, 18, 21, 22] were reported to be satisfactory in different samples. The Cronbach’s alpha for the present study was 0.83.

HHI items #2 and #4 were reworded in 1999 to make the meaning clearer [30]. In the original version, item #2 was “I have short, intermediate and/or long range goals” and item #4 was “I can see a light in the tunnel”. Only two studies that did a factor analysis of the HHI as shown in Table 1 used the new version [21, 22].

### Statistical analysis

SPSS version 22 was used to calculate descriptive statistics and frequency distributions to summarize demographic and clinical characteristics as well as HHI scores.

The application of the Rasch model in order to examine aspects of the HHI’s validity followed a previously described process [31]. The WINSTEPS analysis software program, version 3.69.1.16 [32, 33] was used to conduct the Rasch analysis of the HHI. A Rasch rating scale model was selected as all the items in the HHI are scored on a similar scale.

The Rasch model converts the raw scores from the HHI items simultaneously into item and person equal-interval measures using a logarithmic transformation of the odds probabilities of responses. This computation has been described elsewhere [34]. The converted item calibration measures are then used to evaluate whether items/statements from a scale measure a similar unidimensional construct, viewed as crucial validity aspects of scales in both classical and modern test statistics [34–36]. In a similar manner, the estimated person measures are used to evaluate person response validity and the precision of the scale.

Initially, a differential item functioning (DIF) analysis was performed in order to explore the stability of response patterns of the HHI items in relation to a number of demographic variables, to support evaluation of validity in relation to internal structure and potential unfairness in testing. It is crucial that a scale is not biased in relation to demographic characteristics, as it will otherwise compromise the measures generated, question the validity of the tool, and influence the interpretation of findings. The magnitude of DIF was evaluated using the Mantel-Haenszel statistic for polytomous scales using log-odds estimators [37, 38] in the WINSTEPS program, and DIF with *p* < .01 was considered significant.

The psychometric properties of the HHI rating scale categories were then evaluated with the following criteria: a) a minimum of 10 responses per step category, b) the average measures for each step category should advance monotonically, and c) outfit mean square *(MnSq)* values less than 2.0 for step category calibrations [39].

Evidence of internal scale validity was then investigated by monitoring the item goodness-of-fit statistics. The WINSTEPS program generates mean square (*MnSq*) residuals and standardized *z*-values for each of the twelve HHI items. The goodness-of-fit statistics indicate the degree of match between actual responses on the items and expected responses from the Rasch model. Goodness-of-fit was evaluated focusing on infit statistics, as they are more informative when exploring item fit [27]+ wright 1982. The *MnSq* fit statistic is preferable for item goodness-of-fit with polytomous data as it is less sensitive to sample size [40]. We chose to use a sample-size adjusted criterion [40] for item goodness-of-fit set for infit *MnSq* values between 0.7 and 1.3 logit.

|To detect any additional explanatory dimensions in the data, a *principal component analysis* (PCA) of the residuals was also performed to evaluate the possibility of multidimensionality [41]. The criterion for unidimensionality was set that at least 50% of the total variance should be explained by the first latent variable [42, 43].

Evidence of person response validity was then evaluated by monitoring the person goodness-of-fit statistics. The criterion for evaluating person goodness-of-fit was to reject infit *MnSq* values > 1.4 logit associated with a *z* value > 2 [44, 45]. We also accepted that, by chance, 5% of the sample may fail to demonstrate acceptable goodness-of-fit without a serious threat to validity [45, 46]. We also evaluated the proportion of maximum and minimum scores in the HHI, as this is an indication of ceiling and floor effects, which will also compromise evidence of validity and reliability. We accepted that up to 10% of the sample could demonstrate minimum or maximum scores without a major threat to targeting validity.

Lastly, in order to monitor the precision of the estimated measures, the person separation index was calculated [47]. The separation index reflects the number of statistically different performance strata that the test can identify in the sample, considering the range and precision of the individual person estimates. An index above 1.5 was required to ensure that the HHI could differentiate people with at least two different levels of hope. For the purpose of comparison to more traditional reliability estimates, the Rasch-equivalent Cronbach’s alpha statistic was also reported (person reliability) [47].

## Results

### Sample

A total of 179 patients with pain from bone metastasis were recruited from a university based cancer center and completed the baseline questionnaires including the HHI. Of these, 12 had missing data on the HHI and were excluded from the analysis. The sample’s (*n* = 167) demographic characteristics are summarized in Table 2. Half of the sample (51.4%) was male, and the mean age was 65.5 years. Nearly 40% of the total sample had university or college education, and 20% were living alone. The majority of the patients had breast or prostate cancer, and about a third received chemotherapy or radiation therapy.

**Table 2:** Demographic characteristics of the sample (*n* = 167)

Characteristics	
Age (years) Mean (SD)	65.3 (12.00)
	% (n)
Gender	
Male	52.1 (87)
Female	47.9 (80)
Education	
Primary school	14.5 (24)
Secondary school	45.8 (76)
University/college < 4 years	19.3 (32)
University/college ≥4 years	20.5 (34)
Marital status	
Married/partnered	76.4 (126)
Divorced/separated	8.5 (14)
Unmarried	8.5 (14)
Widowed	6.7 (11)
Living alone	
Yes	19.9 (33)
No	80.1 (133)

*SD* standard deviation

### Rasch analyses

Table 3 shows the mean scores for each individual item for showing the average level of hope in this sample. DIF analyses revealed that three items in the HHI were not functioning in a similar manner in relation to the demographic variables (See Table 4). Item #6 (I feel scared about my future*) demonstrated significant DIF (*p* < .01) in relation to gender (relatively easier to agree with for women as compared to men). Item #3 (Feel all alone) demonstrated significant DIF (*p* < .01) in relation to cohabitation (relatively easier to agree with for participants living alone as compared to participants living with someone). Items #5 (Faith that comforts) and #6 (Scared about the future) also demonstrated significant DIF (*p* < .01) in relation to age (item #5 was relatively easier to agree with for older participants [65 years or older] as compared to younger participants while item #6 was relatively easier to agree with for younger participants as compared to older participants).

**Table 3:** Mean item scores on the Herth Hope Index (*n* = 167)

Individual items	Mean (SD)
1. Positive outlook on life	3.18 (0.58)
2. Presence of goals^a^	3.05 (0.54)
3. Feel all alone	3.54 (0.68)
4. See possibilities in the midst of difficulties^a^	2.90 (0.74)
5. Faith that comforts	2.43 (1.05)
6. Scared about the future	2.41 (0.79)
7. Recall happy/joyful times	3.51 (0.58)
8. Deep inner strength	3.29 (0.54)
9. Give and receive caring/love	3.41 (0.53)
10. A sense of direction	3.11 (0.62)
11. Each day has potential	3.28 (0.56)
12. Life has value and worth	3.37 (0.56)

^a^Items 2 and 4 are reworded (original version of item 2 = I have short, intermediate and/or long range goals, original version of item 4 = I can see a light in the tunnel)

**Table 4:** Evaluation of psychometric properties of the HHI total scale, reduced scale and deleted items (*N* = 167)

Step	Original HHI Scale	Reduced HHI Scale	Deleted items
	(12 items)	(7 items)	(5 items)
	(*N* = 167)	(*N* = 167)	(*N* = 167)
Differential item functioning (DIF): Are item difficulty calibrations stable in relation to key demographic variables?	Gender. Item #6 Cohabitation: Item #3 Age: Items #5 and #6	None	Gender. Item #6 Cohabitation: Item #3 Age: Items #5 and #6
Rating scale functioning: Does the rating scale function consistently across items?	Acceptable	Acceptable	Acceptable
Internal scale validity: Item misfit: How well do the actual item responses match the expected responses from the Rasch model?	Items #3 to #7	None	None
Unidimensionality (i.e., does the scale measure a single construct?): Variance explained by 1st dimension %:	48.3%	52.6%	52.7%
Person-response validity: How well do the individual responses match expected responses from the Rasch model? n (%)	21 (12.4%)	9 (5.3%)	8 (4.7%)
Person misfit, n (%)	4 (2.4%)	15 (8.9%)	8 (4.7%)
Maximum score, n (%)	None	None	None
Minimum score, n (%)			
Person-separation reliability: Can the scale distinguish ≥3 distinct groups of depression in the sample tested? Person-separation index (without extremes)	1.84	1.72	0.82
Person reliability: Cronbach’s alpha equivalent	0.77	0.75	0.40

When evaluating the categorical responses from the HHI items, all set criteria were met. However, the item goodness-of-fit statistics revealed that items #3 and #5 did not meet the criterion set for item goodness-of-fit (see Table 4). By removing both these items, the next iteration revealed that items #4 and #6 also did not meet the criterion for fit and were subsequently removed. In the third iteration, item #7 did not demonstrate acceptable goodness-of-fit to the model and was removed. After the third iteration and the removal of items #3 through #7 (*n* = 5), the remaining seven items on the HHI demonstrated acceptable item fit.

The principal components analyses revealed that the first component explained 52.6% of the total variance in the HHI measures (*n* = 7), which exceeded the criterion of at least 50%.

When evaluating person response validity, 9 of the 167 participants (5.0%) did not demonstrate acceptable goodness-of-fit to the Rasch model in the HHI measures, which met our set criterion of up to 5%. None of the participants had a minimum score (floor effect), and while 15 had a maximum score (ceiling effect), this proportion was less than the set criterion of less than 10%.

The person-separation index for the original HHI scale (*n* = 12) was 1.84, indicating that the HHI is able to separate the participants into at least two distinct strata of hope. The Rasch-equivalent Cronbach’s alpha coefficient for the original HHI scale was 0.77. After deletion of the five HHI items (42%) that demonstrated misfit and/or DIF, the separation index decreased only marginally from 1.84 to 1.72. Thus, we concluded that the reduction of the HHI scale to improve unidimensionality had only a marginal effect on its ability to separate the generated person measures.

As the review of literature on the HHI did not indicate any consistent findings in relation to subdomains of hope across countries or demographics, we decided to explore whether the five items that were deleted from the original 12-item HHI scale demonstrated similar response patterns, indicating a secondary dimension within these items. We therefore combined these five items into a new scale (subdomain) of the HHI. As presented in Table 4, the findings in this 5-item scale were relatively similar to the 7-item scale, except for the unacceptably low separation index. This unidimensional 5-item scale did not separate the sample into any detectable subgroups, which indicates that these generated scale measures are not reliable to use as individual outcomes.

As a final evaluation of the HHI scale, we also evaluated the three suggested subscales in relation to the same aspects and criteria as the total scales. The findings were relatively similar across the subscales and in comparison to the HHI total scale; items #3 and #6 also demonstrated misfit in the subscales. The three subscales also demonstrated even lower levels of separation, indicating that the subscales do not separate the sample into detectable subgroups.

**Fig. 1:**
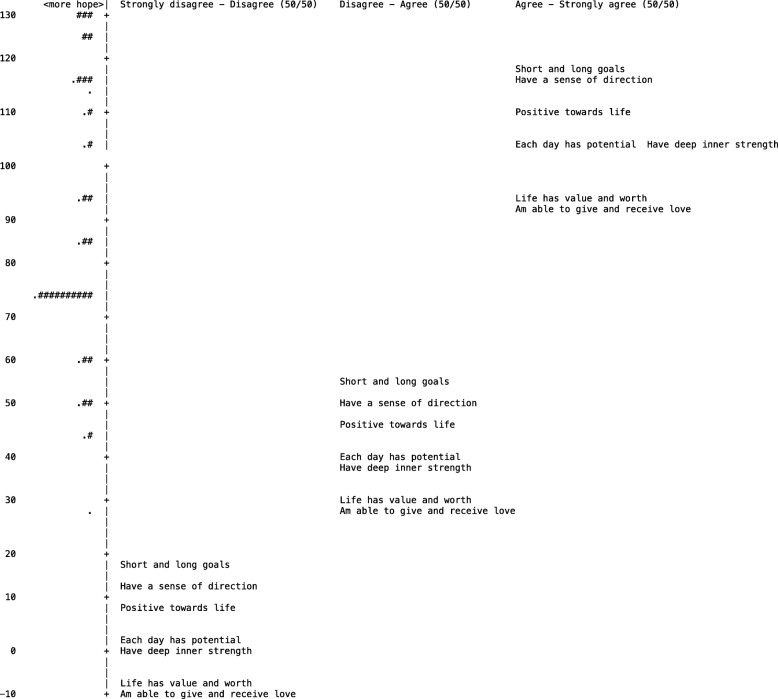
The 7-item version of HHI with each item-category threshold displayed (50/50) in a sample of Norwegian cancer patients (*n* = 179). Each “#” represents five participants

Figure 1 displays the 7-item version of the HHI with each item-category threshold placed along the continuum. Each item-category is positioned where there is a 50/50 probability of marking each of the two categories.

## Discussion

Our study showed that a 7-item version of the HHI, after deleting items #3 through #7, satisfied our criteria for a unidimesional scale and demonstrated evidence of internal scale validity, unidimensionality and person-response validity as well as satisfactory person-separation and person reliability (Table 4). Furthermore, none of the 7 remaining items demonstrated DIFs in relation to gender, age, or cohabitation. In addition, the two negatively worded items (#3 and #6) did not fit the same construct as the 7 HHI items retained after the Rasch analyses. These findings are consistent with other empirical studies using reverse-coded items. Although the use of reversed items has some support in the literature in the development of tests and surveys, and methodological approaches can be applied to deal with some of these issues, the empirical support for such reversed items to contribute to a unidimensional construct is still lacking [48]. The findings from this study therefore supports the approach of not mixing negative and positive items in a scale, as it does not seem to contribute to measurement of a unidimensional construct, at least when applied to the construct of hope.

The item about faith (#5) also failed to demonstrate acceptable fit with the unidimensional construct, indicating more variations in scores on this item than expected in the Rasch model. The role of faith and its relationship to hope likely varies depending on the sample included. In a European survey, it was shown that only 22% of Norwegian citizens responded that “they believe there is a God” [49] compared to 94% in Turkey and Malta. This large variation in faith between countries is a challenge, but it can also be a challenge within a given country, as demonstrated in this study. Faith is also usually defined as a part of hope [14, 15, 50]. The findings from this study indicate that the presence of a faith that comforts (higher scores on item #5) is not associated with higher scores on the other HHI items; people may not experience a faith that comforts but can still have hope, and vice versa.

To control for the possibility of cultural differences, it is also worth comparing our findings to those of other studies performed within Norway. A two-factor solution was found both in the Norwegian general population [8] and in cognitively intact Norwegian nursing home patients [19]. However, the items comprising the different factors are different. Haugen et al. [19] dismissed item #6 (scared about the future) as it was not significant in any models, and showed low reliability. Sweden is culturally linked to Norway, and they also found a two-factor solution [17], but one factor consisted of item #4 and #5, and the rest in the other factor.

The items demonstrating DIF indicate interesting information about subgroup responses and are quite logical in relation to the subgroup characteristics. Even though the observed patterns are logical, it is important to also review such findings from a psychometric and fairness perspective in test construction. As some items are functioning differently for different subgroups, they could theoretically systematically bias the scores/measures from the HHI. It can therefore be important to take action on items demonstrating DIF, e.g., by using split-item techniques. In the case of HHI, this was partially solved by excluding items #3 and #6, which demonstrated DIF and did not fit the underlying construct, as indicated by higher than acceptable goodness-of-fit statistics. The relationship between item misfit and item DIF is indicated in this study, but is not exclusive; see Schulze et al. [51] for somewhat different findings.

The findings from our study do contribute to the diversity of earlier empirical findings of the construct of hope [6, 8, 14, 17, 19–22]. As this was the first time a modern test theory approach (Rasch model) was used, the various findings across validation studies of domains of hope seem to be influenced by several factors, including methodological choices (classical test theory/factor analysis vs. modern test theory/item response theory). As both analytical models impose assumptions that may more or less fit with empirical data [25, 26], the interpretations of findings across studies are complex. One challenge in reducing the HHI scale into a unidimensional construct is that it no longer aligns with the underlying definition of hope [15], or with the basis for the original HHI scale described by the developer [14]. On the other hand, the factor structure varies considerably across empirical studies, in terms of both the number of factors and the items comprising the different factors. The findings from this study further contributes to this diversity. However, the construction of a scale/measure based on a more theoretical or content validity perspective does not always correspond to validity evidence of internal structure from a more measurement perspective [51]. This discrepancy between theory and empiricism in highly relevant concepts for health care is a challenge for clinical research and has not been completely resolved in this study in relation to measuring the concept of hope.

### Methodological limitations

This study has several limitations that need to be considered when reviewing and generalizing the findings. The sample in the present study comprises patients from only one outpatient clinic and all patients had pain from skeletal metastasis. They also all agreed to participate in a randomized controlled trial about pain management, but the data presented here are only from baseline evaluations before the intervention was initiated. We therefore must be cautious about the generalization of our findings to other cancer outpatients. Other Rasch models applied (e.g., a partial credit model) would have imposed fewer assumptions on the data structure, although empirical studies suggest that the choice of a Rasch partial credit model or rating scale model has little impact on the validity findings [52]. Additional validity aspects (e.g., local independence among items; rating scale threshold disordering) should also be considered before making final conclusions of the validity evidence of a given test. There are also other models within modern test theory that could have been used to explore the data further. However, given our review of prior studies, and now also including the findings from this study, the choice of methodological analysis does seem to influence the results of a validity study, and must therefore always be included in the interpretation of empirical findings. The integration of theoretical ideas about a construct, methodological choices/assumptions in our data analysis, and empirical findings from diverse populations/groups, is a complex and critical process that needs to be monitored and discussed consistently in order to support quality control on instrument development.

## Conclusion

A reduced 7-item HHI scale from the present sample demonstrates better psychometric properties (unidimensionality) and a similar level of precision as the original 12-item HHI scale when used with patients with cancer-related pain. None of the original subscales were able to separate the sample into subgroups. However, the identified factor structure has varied across studies. Further empirical research is needed about the dimensionalities potentially embedded in the concept of hope and also to identify differences in hope profiles across items, influenced by patient and/or cultural characteristics. Hope is reported to be an important phenomenon for different patient groups, and measuring hope in the most vulnerable patients is particularly critical. A shorter summative scale that still demonstrates evidence of validity and precision will be easier and less burdensome to use as a screening instrument in clinical practice.

## Abbreviations

DIF: Differential item functioning
HHI: Herth hope index
HHS: Herth Hope Scale
KPS: Karnofsky Performance Status
MnSq: Mean square
PCA: Principal component analysis
US: United States of America
